# A simple method to predict body temperature of small reptiles from environmental temperature

**DOI:** 10.1002/ece3.1961

**Published:** 2016-03-31

**Authors:** Mathew Vickers, Lin Schwarzkopf

**Affiliations:** ^1^ Centre for Tropical Biology and Climate ChangeCollege of Marine and Environmental SciencesJames Cook UniversityTownsvilleQueensland4814Australia; ^2^ CSIROATSIPJames Cook UniversityTownsvilleQueensland4810Australia; ^3^ Oulalab, Centre National de la Recherche ScientifiqueStation d’EcologieExperimentale Moulis2 Route du CNRSMoulis09200France

**Keywords:** copper model, iButton, lizard, operative temperature, thermoregulation

## Abstract

To study behavioral thermoregulation, it is useful to use thermal sensors and physical models to collect environmental temperatures that are used to predict organism body temperature. Many techniques involve expensive or numerous types of sensors (cast copper models, or temperature, humidity, radiation, and wind speed sensors) to collect the microhabitat data necessary to predict body temperatures. Expense and diversity of requisite sensors can limit sampling resolution and accessibility of these methods. We compare body temperature predictions of small lizards from iButtons, DS18B20 sensors, and simple copper models, in both laboratory and natural conditions. Our aim was to develop an inexpensive yet accurate method for body temperature prediction. Either method was applicable given appropriate parameterization of the heat transfer equation used. The simplest and cheapest method was DS18B20 sensors attached to a small recording computer. There was little if any deficit in precision or accuracy compared to other published methods. We show how the heat transfer equation can be parameterized, and it can also be used to predict body temperature from historically collected data, allowing strong comparisons between current and previous environmental temperatures using the most modern techniques. Our simple method uses very cheap sensors and loggers to extensively sample habitat temperature, improving our understanding of microhabitat structure and thermal variability with respect to small ectotherms. While our method was quite precise, we feel any potential loss in accuracy is offset by the increase in sample resolution, important as it is increasingly apparent that, particularly for small ectotherms, habitat thermal heterogeneity is the strongest influence on transient body temperature.

## Introduction

With the threat of climate change, thermal ecology studies have never been more urgent. Recent studies demonstrate the importance of understanding thermal heterogeneity at a fine scale (Sears et al. 2011; Sears and Angilletta 2015) while historically, highest importance has been placed on precise and accurate body temperature prediction (Porter and Gates 1969). Accordingly, studies typically describe the thermal quality of the environment in terms of “operative environmental temperature” (*T*_*e*_): the steady‐state temperature of an object with the same size and shape as the focal organism, with zero heat capacity. The “object” used to measure *T*_*e*_ ranges from detailed physical models that mimic the organism made of copper or plastic, to simple copper tubes, PVC tubes, iButtons, HOBOs, and Tidbits (Bakken 1976; Hertz et al. 1993; Vitt and Sartorius 1999; Dzialowski and O'Connor 2001; Shine and Kearney 2001; Dzialowski 2005). Alternatively, complex mathematical models predict body temperature from first principles or rely on high‐dimensional data sets collected by numerous sensors (Christian and Weavers 1996; Kearney 2006; Fei et al. 2012a; Barton et al. 2014). We aim to improve the accessibility of predicting body temperature of small reptiles using a simple and cheap method.

Our method directly estimates body temperature of small reptiles from temperature data collected by simple temperature sensors (DS18B20^™^, Maxim Integrated Products) and transformed using a single parameter heat transfer equation. By comparison with previously used methods, ours is simple to construct and use. At ca. $US1 per sensor, it is also incredibly cheap compared to cast or printed models (Watson and Francis 2015) or other thermal sensors, making it accessible to researchers regardless of funding situation. The accessibility of our method means thermal structure can be mapped cheaply and comprehensively at a fine scale which is of increasing interest as the importance of transient body temperatures and habitat thermal structure eclipses that of steady state, equilibrium, or operative body temperature (O'Connor 1999; Whitaker and Shine 2002; Seebacher et al. 2003; Seebacher and Shine 2006; Fei et al. 2012b).

With our method, we do not attempt to explain the role or importance of convection, conduction, and irradiation on body temperature. We aim simply to improve accessibility to body temperature predictions. Highly complex models are concomitant with an increase in number and types of recorders, programming time, and mathematical acuity required. High mathematical complexity can make a model inaccessible, while demand for difficult, numerous, or expensive sensors and models can limit sample size in both space and time. For example, very detailed model may partition the effect of many sources of radiation (Fei et al. 2012a) as well as physiology and posture (Stevenson 1985) on body temperature. However, the practical use of such models may be limited, as not all variables can be measured comprehensively in the field. On the other hand, highly integrative models cannot necessarily predict the effect of fine‐scale landscape heterogeneity (Kearney et al. 2013) on thermoregulation.

Our simple model relies on few inputs, simple temperature recorders, and low computational overheads. Cheaper temperature recorders means more temperature recorders: important in understanding thermal structure and heterogeneity, possibly the most important aspect of the thermal environment to small ectotherms (Sears and Angilletta 2015). Currently, simple models exist for predicting the transient body temperatures of large ectotherms, whereas models for predicting the body temperatures of small‐bodied ectotherms tend to be highly complex (O'Connor 2000; Seebacher and Shine 2004).

We demonstrate our model in the laboratory and field, test its accuracy against real lizard body temperature, and demonstrate optimization of the only model parameter, *K*, which relates the size and mass of the sensor/physical model to the size and mass of the organism being modeled. We compare our model's performance with T_e_ collected using classic copper models (ca. US$3.50 per model for 100, plus a temperature sensor and ca. 30 h effort/model, Watson and Francis 2015), Thermochron iButtons^™^ (ca. US$21.00 per iButton, Maxim Integrated Products) and naked DS18B20 temperature sensors (ca. US$1.00/sensor, plus US$ 1.00/m cable, plus US$50.00 Raspberry Pi = US$2.50 per sensor). Any logging computer such as an Arduino could be used in place, of the Raspberry Pi. All three methods used the same, DS18B20, temperature sensor which records temperature with 12‐bit precision.

## Methods

### Study species

Experiments were conducted at three locations in Queensland, Australia: Lizard Island Research Station (September 2013, 14.66°S, 145.55°E), Wambiana Station near Charters Towers (October 2013, 20.55°S, 146.10°), and Townsville (19.2564° S, 146.8183° E). At Lizard Island, the body temperatures of sandy rainbow skinks (*Carlia dogare*, Covacevich and Ingram, 1975, *n* = 4, SVL 48 mm, mass 4.8 g) were compared to temperatures collected using iButtons^™^. At Wambiana, the body temperatures of shaded‐litter rainbow skinks (*Carlia munda*, De Vis 1885, *n* = 3, 45 mm, 4.1 g) were compared with temperatures recorded by DS18B20 sensors and copper models fitted with DS18B20 sensors, and in Townsville, body temperatures of lined rainbow skinks (*C. jarnoldae*,* n* = 4, 45 mm, 4.0 g) were compared to iButtons wrapped in undyed cotton bags (neutral beige color), DS18B20 sensors and copper models in a natural (outdoor) setting. In all locations, lizards were captured by hand and returned to the laboratory, where experiments were conducted immediately. Lizards were all returned to their point of capture within an hour.

### Experimental design

#### Lizard Island and Wambiana

Lizards were taped to an unstained block of pine (300 mm L × 50 mm H × 100 mm W) using Millipore tape, and a temperature recorder was placed immediately adjacent to them. The recorder was either an iButton^™^ in a 3 × 3 cm “calico” (equal weft and warp plain weave fabric in unbleached cotton) cloth bag for consistency with other studies (Vickers et al. 2011) or both a sensor (DS18B20) and a copper model. Copper models were simple hollow tubes of 0.2 mm copper sheet, 1.0 cm diameter at the head end, 7 cm long, tapered to the tail, and spray‐painted a neutral brown to match the reflectance of the lizards (confirmed by an Ocean Optics USB2000+ spectrometer, 200–880 nm range, 4.1 nm resolution) Thermal sensors, DS18B20, were placed in the thorax of the model. The apparatus was placed 300 mm directly below a 60W light bulb until the lizard showed early signs of distress (struggling, and high body temperature, <38°C, usually after approximately 3–4 min) and was then removed from under the light bulb to allow cooling. Temperatures were recorded for another 3–5 min after the apparatus was removed from the direct radiation. All experiments took place in rooms cooled to approximately 23°C. Lizards were not run multiple times nor for a longer period in order to keep thermal stress to a minimum, but in particular with the outdoor experiments, the experiment represents the one cycle of the possible thermal range a lizard will experience.

#### Townsville

A similar wooden‐block apparatus was used in Townsville, but instead of laboratory conditions, the wooden block was placed outdoors. The block held one lizard, one wrapped iButton, one Copper model, and one DS18B20 sensor. The experiment was run over 2 days with variable wind and cloud conditions in May 2015. Each lizard began in the shade, on the ground. After approximately 4 min, the apparatus was moved into the open, remained there until the body temperature of the lizards reached 38.5°C (although body temperatures occasionally reached 40°C due to thermal inertia), and the apparatus was returned to the shade until lizard body temperature stabilized again.

### Temperature measurement

#### Models

iButtons^™^ recorded temperatures every minute, and sensor and copper models recorded temperatures every 40 sec.

The DS18B20 sensors, used both as naked sensors and in the copper models, were attached to a Raspberry Pi^™^ computer by a 10‐m Category 5 Ethernet cable. The sensors were attached as shown in Appendix S1 and were controlled by a purpose‐written script that polled the sensors every 40 sec.

#### Lizards

A calibrated thermal probe (a type‐K thermocouple, 3‐mm gauge) was inserted in the lizard's cloaca and attached to a quick‐reading digital thermometer (Comark KM‐C28K), and body temperatures were recorded to the nearest 0.1°C every minute. For each lizard, a GAM (generalized additive model) was fitted to temperatures recorded over time using a cubic regression spline smoother, in function MGCV in R (Wood 2011; R Core Team 2014). The GAM was used to predict lizard body temperature every second, and all GAMs had near‐perfect fit, total deviance explained > 99.5%. The heterogeneity of variance in the models was minor enough as to be unlikely to cause errors in interpretation. GAMs were fitted following (Zuur et al. 2009) and (Wood 2011).

### Modeling and optimization

Lizard body temperature was predicted from model and iButton temperature recordings in Equation (1) (adapted from (Mitchell 1976; Angilletta 2009). From the literature, the range of *K* for a 4.5 g skink standing approximately 1.0 cm above the substrate is in the range of 0.002–0.7 (Mitchell 1976). The maximum *K* value used was 0.2, as predictions with *K* values beyond this were higher than observed body temperatures. To determine the value of K that best estimated transient body temperature for *Carlia*, the equation was run using all K values in a sequence from 0.02 to 0.2, incremented by 0.01, the results of which were used for comparison with the real lizards paired with the recorder.

Root‐mean‐square error (RMSE) between operative environmental temperature estimates and the lizard GAM determined the “best” *K* value (i.e., the one with the lowest RMSE) for closest model fit. Visual inspection of plots was also used in conjunction with sum of squared differences to determine the “best” *K* value for estimating equilibrium temperature and rate of increase or decrease of body temperature.

Equation (1). Predicted body temperature, *T*_*b*_, at time *i*, using body temperature at time *i*−1, $T_{b_{\left(i-1\right)}}$, operative environmental temperature (*T*_*e*_) at time *i*, time spent at temperature $T_{e_{\left(i\right)}}$ (*t*), and a parameter, *K*, a thermal time constant that incorporated body size. Estimated body temperature was then used as $T_{b_{\left(i-1\right)}}$ in the next iteration. In this way, body temperature was integrated over time and would only reach equilibrium with the site if the site remained at one *T*_*e*_ for long enough.

(1) $$T_{b_{\left(i\right)}}=T_{e_{\left(i\right)}}+\left(T_{b_{\left(i-1\right)}}-T_{e_{\left(i\right)}}\right)\times \exp\left(-Kt\right).$$

## Results

**Figure 1:**
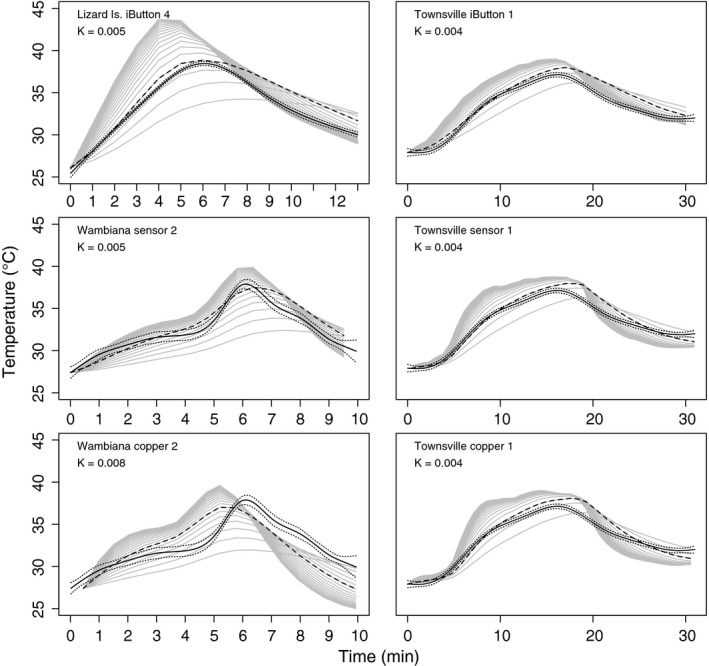
Effect of varying *K* in body temperature prediction from model temperatures (iButton, copper model, DS18B20 sensor) in laboratory (Lizard Is., Wambiana) and field (Townsville) conditions. One plot per model/lizard pair. Topmost gray line is sensor temperature, successive gray lines moving downward are body temperature predictions using increasing *K* values (from 0.002 to 0.02), and each line is one *K* value. Measured lizard body temperature is shown (black line), with GAM prediction intervals (dotted, ±2*SE). The *K* value with the lowest RMSE between predicted and actual lizard body temperature is indicated and drawn as a dashed line.

**Figure 2:**
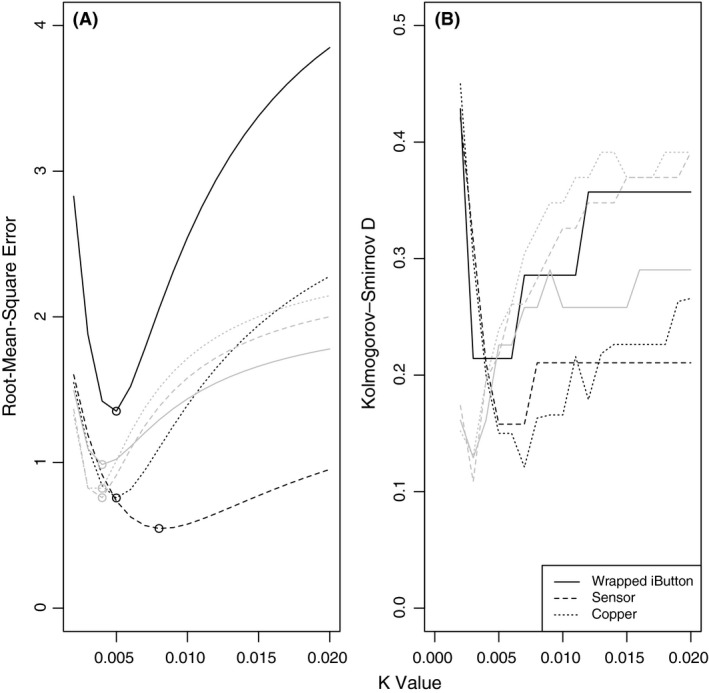
(A) Change in accuracy (lower RMSE = higher accuracy) with increasing *K* for three sensor temperatures in laboratory (Lizard Is., Wambiana, black) and field (Townsville, gray) conditions. Points indicate lowest RMSE value. (B) Response of the Kolmogorov–Smirnov *D* for difference in distribution shape between predicted and actual lizard body temperature for each *K* value. The lowest *D*‐values indicate the highest similarity between distribution of cloacal temperature and modeled temperature.

Body temperature of *Carlia* was well predicted by either of the three models (wrapped iButton, naked DS18B20 sensor, and copper models, Figs. 1, 2, Appendices S2–7). The optimal value of the parameter *K* differed slightly among model types, although was most consistent for naked DS18B20 sensors. Parameter *K* ranged from 0.003 to 0.006 for iButtons, 0.004 to 0.008 for copper models, 0.004 to 0.005 for naked sensors (Appendices S2–7). The cheapest model depends on the number of models used: for fewer than 3, iButtons were cheaper; for more, DS18B20 with Raspberry Pi was cheaper.

### Under laboratory conditions

*K* values best predicting lizard body temperature from iButton temperatures under laboratory conditions differed slightly among the four *C. dogare* from Lizard Island, and also slightly among the four *C. decora* from Townsville (Figs. 1, 2, Appendices S2, S3). Lowest total difference measured as lowest RMSE (root‐mean‐square error), between predicted and actual lizard body temperature varied from 0.48 to 1.42 among lizards, at 0.003 < *K* < 0.006 (Appendix S2). Particular aspects of lizard body temperature were optimized at slightly different, overlapping, *K* values: The best estimate of maximum temperature was for *K* = 0.004–0.009 (nearest peak temperature); of increasing body temperatures (increasing slope) was near *K* = 0.005; and of decreasing body temperature (decreasing slope) was higher, near 0.01–0.015 (Appendices S2, S3). There were no significant differences between actual lizard temperatures and predicted body temperatures using optimal *K* estimated from iButtons^™^ according to Kolmogorov–Smirnov tests, although the test statistic, *D*, indicated that the *K* value that yielded the prediction least different from actual body temperature was near 0.005 for all lizards (Appendix S3).

Model RMSE for both the copper models and the sensor models under laboratory conditions were less variable than model outputs from iButtons^™^, although optimal *K* was slightly more variable. For the copper models, lowest RMSE ranged from 0.75 to 0.9, at *K *=* *0.004–0.008, RMSE was lower still for the DS18B20, ranging from 0.32 to 0.69 at *K *=* *0.004–0.008 (Appendices S4, S5).

Particular aspects of lizard body temperature were best estimated using the same *K* parameters, among lizards using either copper model or naked DS18B20 sensors: best estimates of maximum, increasing and decreasing body temperature for Copper models was approximately *K* = 0.005, and for naked sensors was *K* = 0.004 (Appendices S4, S5).

There were no significant differences between actual lizard temperatures and predicted body temperatures using optimal *K* estimated from DS18B20 sensors or copper models according to Kolmogorov–Smirnov tests, the test statistic, *D*, indicated that the *K* value yielding the prediction least different from actual body temperature was near 0.005–0.007 for copper models and 0.003–0.005 for sensors (Appendix S5).

### Under field conditions

As with laboratory conditions, parameter K was most variable for iButtons (Appendices S6, S7). For iButtons, RMSE varied between 0.42 and 1.13, at *K* = 0.004–0.008; for copper models, RMSE 0.56 < RMSE < 0.96 at 0.003 < *K* < 0.004; DS18B20 sensors were consistently more similar to lizard body temperature with 0.51 < RMSE < 0.76 at 0.003 < *K* < 0.004. There was no significant difference between any model temperature and the associated lizard body temperature. Naked sensors and copper models performed similarly in terms of similarity to real lizard temperature, and both outperformed iButtons (Appendices S6, S7).

## Discussion

We tested three model types (wrapped iButtons, copper models, and naked DS18B20 sensors) for accuracy in predicting *Carlia* lizard body temperatures in laboratory and field conditions and found that naked DS18B20 sensors were the most accurate as well as cheapest. We used a simple heat transfer equation to transform operative temperature to lizard body temperature, and it is clear that a given system must be calibrated to lizard size, although it seems unimportant whether this is done under field or laboratory conditions. Accuracy scored by RMSE was better for all three models used here than other methods reported in the literature (Fei et al. 2012a), indicating that for our lizards, there was no sacrifice in model accuracy when our simple model was employed. This is similar to previous findings that compare simple models (Vitt and Sartorius 1999). We understand the limitations in our model in terms of interpreting the relative contribution of multiple sources of radiation, water loss, wind speed, posture etc. that more realistic looking models allow (Shine and Kearney 2001; Bakken and Angilletta 2014; Barton et al. 2014); however, our goal was to simply and accurately estimate body temperature in a way that allows use of cheap, accessible technology. Our method provides high‐resolution detail on transient body temperature, although the *K* value used should be determined for each size of organism and sensor (Bakken and Angilletta 2014). For *Carlia* skinks, the best estimation of body temperature from iButtons^™^ occurred when we used a *K* value between 0.003 and 0.008, while for the DS18B20 sensors and copper models, best estimation occurred when we used a *K* value near 0.003–0.005. However, estimations from iButtons^™^ were more variable than from the DS18B20 sensors, which may be due to slight inconsistencies in iButton^™^ manufacture, in the cloth they were wrapped with, or in their positioning. There was no indication of bias in body temperature estimation from any model, in some cases, the model was overestimated, and in other cases, it was underestimated observed lizard body temperature (Fig. 1, Appendices S2, S4). As with iButtons, variation may be due to slight positioning differences or physical differences among models, although much of the variation is likely due to individual size differences and perhaps interindividual differences in physiological and metabolic goals or ontogeny.

Our model can also use data collected for other studies, for example, using simple sensors (Hertz et al. 1993; Bauwens et al. 1996; O'Connor 2000; Dzialowski 2005) and iButtons (Aubret and Shine 2010; Besson and Cree 2010). Further subsampling temperature data collected by an array of sensors can be used to simulate an ectotherm moving in the habitat as any body temperature prediction is based on previous body temperature, previous environment temperature, new environment temperature, and the time to transit. This means our method can be used to test hypotheses about the importance of transient body temperature in different habitat structures (e.g., Sears et al. 2011). Similarly, the model could use historical data, improving our understanding of climate change so far on small ectotherms body temperature.

Naked DS18B20 sensors, that is, sensors without a surrounding copper model or iButton casing, yielded the most accurate predictions of lizard body temperature, and at ca. US $1 are a cost‐effective alternative to copper models for estimating body temperature of field active ectotherms. The most portable method was iButtons, and their independent power and operation means they are suited to any terrain, although they were more labor intensive to deploy, collect, and download than the other methods. Wiring batches of DS18B20 sensors carries an initial time overhead and can make deployment (a 10‐m cable) unpleasant in some habitats, although they automatically record to a database, reducing potential human error in labeling and downloading sensors.

Our method has some limitations: We used very simple copper models by comparison with some previous studies (Hertz 1992; Bakken and Angilletta 2014). There is, however, a limited return rate of accuracy in operative temperature estimates for increased precision of copper models: Adding physical structure or color to models can have less influence on predicted temperature than placement (Shine and Kearney 2001). In light of this, we feel that using many simple, cheap models to characterize the thermal environment provide better information than highly precise temperature estimates at few, specific, locations. This is particularly so for small motile organisms who move relatively fast and have low thermal inertia. Our model was tested in controlled and field conditions similar to those employed by Shine and Kearney (2001), and it is clear many factors such as convection, conduction, and posture affect body temperature, although we make no attempt to interpret that, and the magnitude of such effects may be small for small ectotherms (Fei et al. 2012b; Barton et al. 2014). And lastly, our method covers only one temperature cycle (cool – hot – cool), and further study into long‐term temperature prediction over multiple hot–cold cycles may help to improve our equation or prediction intervals.

We argue that the increased precision of description of habitat thermal heterogeneity allowed by high‐resolution sampling of microhabitats using cheap systems will allow detailed analysis of the importance of spatial and temporal thermal structure. This may have some advantage in understanding the importance of thermal structure relative to other features of the biotic and abiotic environment in comparison to predictions of steady‐state (equilibrium) body temperatures that seem currently favored (Adolph 1990; Hertz 1992; Dias and Rocha 2004; Fei et al. 2012b; Bakken and Angilletta 2014).

Our method uses a simple heat transfer equation and common, easily available, and cheap environmental temperature measuring techniques. The method can be used to estimate transient body temperatures of a small lizard moving dynamically through a complex habitat. The utility of this method is threefold: Estimated body temperatures are easy to calculate, it is easy to collect large amounts of data, and it is possible to use previously collected data to estimate transient body temperatures.

## Data Accessibility

The data are stored in Datadryad, http://dx.doi.org/10.5061/dryad.23dn4

## Conflict of Interest

The authors have no conflict of interests to declare.

## Supporting information


**Appendix S1.** Connection of the DS18B20 temperature sensor to the Raspberry Pi GPIO board was as shown, with a pull‐up resistor connecting the data and power.
**Appendix S2.** *Carlia dogare* body temperature predicted by iButton^™^ at Lizard Island: one plot per lizard/iButton^™^ pair.
**Appendix S3.** (a) Response of RMSE difference between predicted and actual *Carlia dogare* body temperature (shown in Figure 1) to variations in the *K* value. Points indicate the *K*‐value with the lowest RMSE. One line per lizard/iButton pair. (b) Response of the Kolmogorov–Smirnov D for difference in distribution shape between predicted and actual *Carlia dogare* body temperature for each *K* value. The lowest *D*‐values indicate the highest similarity between cloacal temperature and modelled temperature.
**Appendix S4.** *Carlia munda* body temperature predicted by iButton^™^ at Wambiana: one plot per lizard/sensor pair, each row is a unique lizard, left column DS18B20 sensor, right column copper model. Topmost grey line is an iButton^™^ temperature, successive grey lines moving downward are body temperature predictions using increasing *K* values (from 0.002 – 0.02), each line is one *K* value.
**Appendix S5.** (a) Response of RMSE difference between predicted and actual *Carlia munda* body temperature (shown in Figure 3) to variations in the *K* value. Points indicate the *K*‐value with the lowest RMSE. One line per lizard/iButton pair. (b) Response of the Kolmogorov–Smirnov D for difference in distribution shape between predicted and actual *Carlia munda* body temperature for each *K* value, copper model (grey), DS18B20 sensor (black).
**Appendix S6.** *Carlia dogare* body temperature predicted from 3 sensors, in columns: (a) iButton^™^, (b) copper models, and (c) naked DS18B20 sensors at Townsville; one plot per lizard/sensor pair, each row a unique lizard.
**Appendix S7.** (a) Response of RMSE difference between predicted (one line per model) and actual *Carlia dogare* body temperature (shown in Figure 5) to variations in the K value. Points indicate the K‐value with the lowest RMSE. One line per lizard/model pair, one plot per lizard. (b) Response of the Kolmogorov‐Smirnov D for difference in distribution shape between predicted and actual *Carlia dogare* body temperature for each K value, one line per lizard/model pair, one plot per lizard.
