# Supplementary material for: A simple method to predict body temperature of small reptiles from environmental temperature
Source: Ecol Evol. 2016 Mar 31;6(10):3059–66. doi: 10.1002/ece3.1961 (PMC4870193; doi:10.1002/ece3.1961)
Supplement: Supplementary file 1 — Appendix S1. Connection of the DS18B20 temperature sensor to the Raspberry Pi GPIO board was as shown, with a pull‐up resistor connecting the data and power. Appendix S2. Carlia dogare body temperature predicted by iButton™ at Lizard Island: one plot per lizard/iButton™ pair. Appendix S3. (a) Response of RMSE difference between predicted and actual Carlia dogare body temperature (shown in Figure 1) to variations in the K value. Points indicate the K‐value with the lowest RMSE. One line per lizard/iButton pair. (b) Response of the Kolmogorov–Smirnov D for difference in distribution shape between predicted and actual Carlia dogare body temperature for each K value. The lowest D‐values indicate the highest similarity between cloacal temperature and modelled temperature. Appendix S4. Carlia munda body temperature predicted by iButton™ at Wambiana: one plot per lizard/sensor pair, each row is a unique lizard, left column DS18B20 sensor, right column copper model. Topmost grey line is an iButton™ temperature, successive grey lines moving downward are body temperature predictions using increasing K values (from 0.002 – 0.02), each line is one K value. Appendix S5. (a) Response of RMSE difference between predicted and actual Carlia munda body temperature (shown in Figure 3) to variations in the K value. Points indicate the K‐value with the lowest RMSE. One line per lizard/iButton pair. (b) Response of the Kolmogorov–Smirnov D for difference in distribution shape between predicted and actual Carlia munda body temperature for each K value, copper model (grey), DS18B20 sensor (black). Appendix S6. Carlia dogare body temperature predicted from 3 sensors, in columns: (a) iButton™, (b) copper models, and (c) naked DS18B20 sensors at Townsville; one plot per lizard/sensor pair, each row a unique lizard. Appendix S7. (a) Response of RMSE difference between predicted (one line per model) and actual Carlia dogare body temperature (shown in Figure 5) to variations in the K value. Poin [file ECE3-6-3059-s001.docx]

## Supporting Information and Appendices

Appendix S1. Connection of the DS18B20 temperature sensor to the Raspberry Pi GPIO board was as shown, with a pull-up resistor connecting the data and power. Pin 1 and 3 can be moved to any Power and Ground on the GPIO respectively. At this time, one-wire data transfer is limited to GPIO 4.

| DS18B20 Pin | Raspberry Pi GPIO | | |
| --- | --- | --- | --- |
| 1 | 13 | Ground |  |
| 2 | 4 |  | 4.7 kΩ resistor between these |
| 3 | 1 | 3.3V Power |  |

Appendix S2. *Carlia dogare* body temperature predicted by iButton™ at Lizard Island: one plot per lizard/iButton™ pair. Topmost grey line is an iButton™ temperature, successive grey lines moving downward are body temperature predictions using increasing K values (from 0.002 – 0.02), each line is one K value. Measured lizard body temperature is shown (black line), with GAM prediction intervals (dotted, ± 2*SE). The K value with the lowest RMSE between predicted and actual lizard body temperature is indicated and drawn as a dashed line.

Appendix S3. (a) Response of RMSE difference between predicted and actual *Carlia dogare* body temperature (shown in Figure 1) to variations in the K value. Points indicate the K-value with the lowest RMSE. One line per lizard/iButton pair.

(b) Response of the Kolmogorov-Smirnov D for difference in distribution shape between predicted and actual *Carlia dogare* body temperature for each K value. The lowest D-values indicate the highest similarity between cloacal temperature and modelled temperature.

Appendix S4. *Carlia munda* body temperature predicted by iButton™ at Wambiana: one plot per lizard/sensor pair, each row is a unique lizard, left column DS18B20 sensor, right column copper model. Topmost grey line is an iButton™ temperature, successive grey lines moving downward are body temperature predictions using increasing K values (from 0.002 – 0.02), each line is one K value. Measured lizard body temperature is shown (black line), with GAM prediction intervals (dotted, +-2*SE). The “best” K value (i.e., the one with the lowest RMSE between predicted and actual lizard body temperature) is indicated and drawn as a dashed line.

Appendix S5. (a) Response of RMSE difference between predicted and actual *Carlia munda* body temperature (shown in Figure 3) to variations in the K value. Points indicate the K-value with the lowest RMSE. One line per lizard/iButton pair.

(b) Response of the Kolmogorov-Smirnov D for difference in distribution shape between predicted and actual *Carlia munda* body temperature for each K value, copper model (grey), DS18B20 sensor (black).

Appendix S6. *Carlia dogare* body temperature predicted from 3 sensors, in columns: (a) iButton™, (b) copper models, and (c) naked DS18B20 sensors at Townsville; one plot per lizard/sensor pair, each row a unique lizard. Topmost grey line is a recorded model temperature, successive grey lines moving downward are body temperature predictions using increasing K values (from 0.002 – 0.02), each line is one K value. Measured lizard body temperature is shown (black line), with GAM prediction intervals (dotted, +-2*SE). The K value with the lowest RMSE between predicted and actual lizard body temperature is indicated and drawn as a dashed line.

Appendix S7. (a) Response of RMSE difference between predicted (one line per model) and actual *Carlia dogare* body temperature (shown in Figure 5) to variations in the K value. Points indicate the K-value with the lowest RMSE. One line per lizard/model pair, one plot per lizard.

(b) Response of the Kolmogorov-Smirnov D for difference in distribution shape between predicted and actual *Carlia dogare* body temperature for each K value, one line per lizard/model pair, one plot per lizard.
